# A Randomised Controlled Trial Comparing Chemomechanical (Carie-Care™) Versus Conventional Caries Removal for Atraumatic Restorative Treatment

**DOI:** 10.1155/tswj/6689053

**Published:** 2025-01-30

**Authors:** Saloni Gupta, Kalyana Chakravarthy Pentapati, Shashidhar Acharya

**Affiliations:** Department of Public Health Dentistry, Manipal College of Dental Sciences, Manipal Academy of Higher Education, Manipal 576104, Karnataka State, India

## Abstract

**Objective:** This study aimed to evaluate and compare the clinical effectiveness of chemomechanical caries removal (CMCR) using Carie-Care™ versus conventional caries removal for atraumatic restorative treatment (ART).

**Methods:** The study included 32 children aged 6–15 years with one or more one-surface cavitated carious lesions on the occlusal surface of permanent dentition. A total of 82 restorations were placed in permanent molars. The teeth were randomly assigned to two groups and monitored for 18 months after the intervention. For Group 1, Carie-Care™ gel was applied directly to the carious lesion, followed by cavity washing and gentle excavation using hand instruments. For Group 2, caries was removed using the mechanical method only. The success rate of the restorations, as well as the time taken and pain reactions measured by the sound eye motor (SEM) scale, were assessed between the two groups.

**Results:** The SEM scores were significantly higher (*p*  <  0.05) in the conventional ART group than in the Carie-Care™ group. The mean time taken for caries removal in the Carie-Care™ group (731.15 ± 197.48 s) was significantly higher than in the ART group (596.66 ± 158.96 s) (*p* < 0.001). However, there was no significant difference in the clinical performance of Type IX restoration between the groups (*p*=0.69).

**Conclusions:** The success rates of the restorations were similar between the two methods. However, the added advantage of less trauma associated with using a chemomechanical agent such as Carie-Care™ makes it an attractive option for community health and school dental programs.

## 1. Introduction

Atraumatic restorative treatment (ART) is a minimally invasive technique that involves the manual removal of infected carious structures from the tooth, followed by the placement of an adherent filling material, typically glass-ionomer Cement (GIC) [1]. Initially developed to address the dental care needs of underserved rural communities, ART is now being recognised for its applicability in diverse populations in both developed and developing countries. The previous research has demonstrated its clinical success and patient acceptability, particularly among children, the elderly and individuals with disabilities [2–4].

For the restoration of cavities using ART, GIC is universally recommended. High-viscosity GICs have showed superior mechanical and physical properties in Class I restorations, making them particularly suitable for ART [5, 6]. ART has been well received in community field settings, with most patients reporting minimal pain during instrumentation, though deeper carious lesions near the pulp may elicit higher levels of discomfort. Moreover, single-surface ART restorations have exhibited better survival rates compared to multisurface restorations [7–9].

Chemomechanical caries removal (CMCR) is another conservative method for caries removal. It involves the application of a gel containing an amino acid solution and hypochlorite, which dissolves collagen fibres degraded by the caries process, facilitating their manual removal. A recent material developed for CMCR is Carie-Care™ [10], which contains purified papain enzyme with demonstrated antibacterial and anti-inflammatory properties. The addition of clove oil provides analgesic and antiseptic effects whilst minimising adverse effects on noncarious tissues. When compared to conventional ART, CMCR has been found to be equally efficient in caries removal, albeit taking more time [11].

To date, there is a dearth of studies comparing the clinical effectiveness of chemomechanical versus conventional caries removal for ART in permanent teeth. In addition, no literature has reported on the difference in pain perception experienced by patients whilst undergoing restorative therapy between ART and CMCR groups using the sound eye motor (SEM) scale. This study aimed to evaluate and compare the clinical effectiveness of CMCR using Carie-Care™ versus conventional caries removal for ART. The success rate of the restorations as well as the time taken and pain reactions measured by the SEM scale were assessed between the two groups. The null hypothesis assumed no significant differences between the two methods in terms of effectiveness, time taken and pain reaction.

## 2. Methodology

The trial was conducted among children from five schools in Udupi district, Karnataka state, India, from January 2020 to September 2021. Children with one or more one-surface cavitated carious lesions on the occlusal surface in permanent dentition, accessible to hand instruments, were included. Those with specific contraindications, such as the presence or history of pain/abscess/fistula/swelling near the carious tooth, deep carious lesions exposing pulp, tenderness on percussion, syndromes, ongoing orthodontic treatment, developmental defects of teeth or other conditions hindering caries excavation, were excluded. Informed consent was obtained from both the children and their parents, and the study was approved by the Kasturba Medical College and Kasturba Hospital Institutional Ethics Committee (IEC: 846/2019). The protocol was registered with the Clinical Trial Registry of India (CTRI/2020/01/023046).

The sample size was estimated using G-power software (3.1.9.7. version) based on an effect size of 0.5, a power of 95%, and a significance level (*α*) of 0.05 yielded a sample size of 60 teeth. The effect size of 0.5 was based on the results obtained by Kumar et al. [11] who reported a difference in clinical efficacy of 55% between Carie-Care^TM^ and control groups. Considering a potential attrition rate of 20%, the sample size was inflated to 75 teeth. The researchers conducted the study with a single-blinded parallel design where trial participants were blinded, and their teeth randomised into two groups using block randomisation.

**Figure 1:**
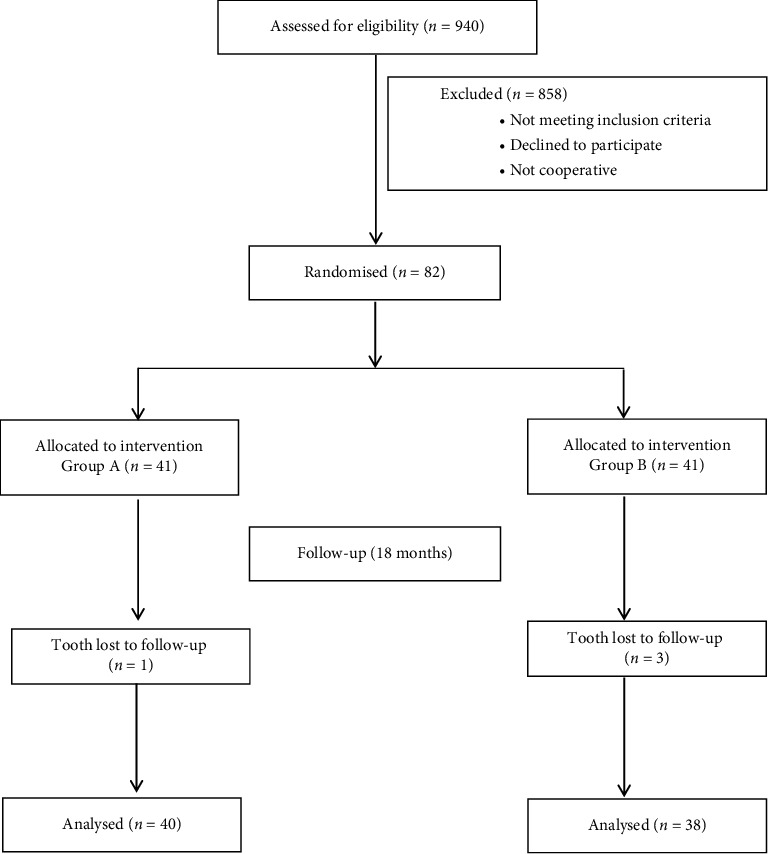
Flowchart of the study protocol.

**Figure 2:**
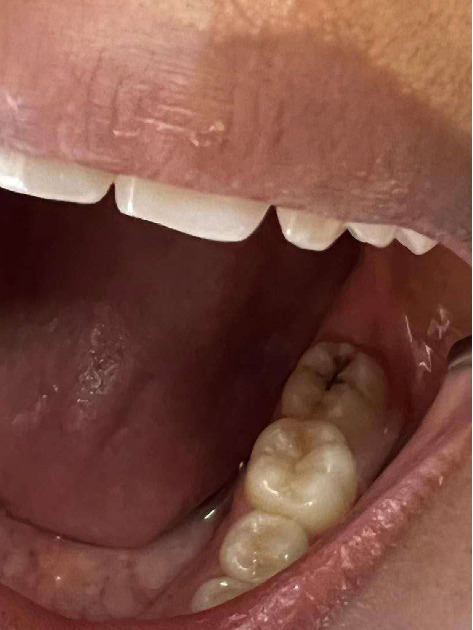
Caries lesion on lower molar.

**Figure 3:**
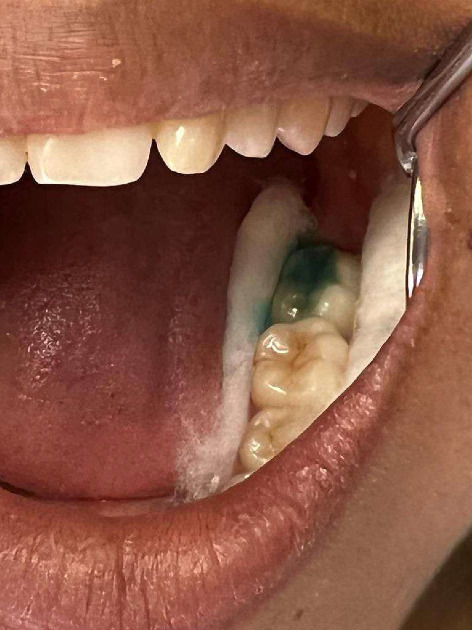
Lesion postapplication of Carie-Care.

**Figure 4:**
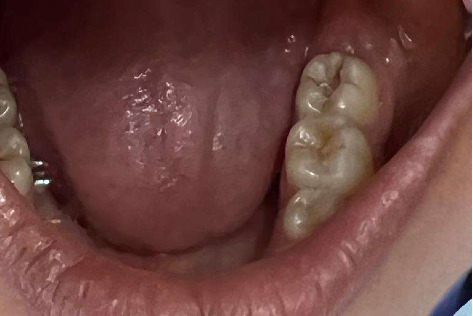
Lesion postexcavation of caries.

**Figure 5:**
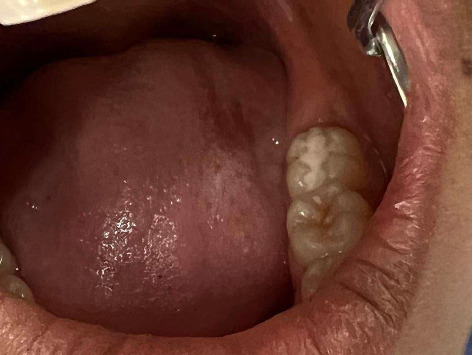
Tooth after restoration.

The procedure was performed on the school campuses, and the eligible participants were seated on chairs facing windows or doors to ensure natural light. The primary investigator enroled the participants, and two examiners who were trained and calibrated in ART evaluation, screened 940 children aged between 6 and 12 years in natural lighting conditions, identifying 208 eligible participants. After providing participant information sheets and informed consent forms to parents, 160 children were excluded due to lack of parental consent, and 16 children were further excluded due to noncooperation or absence. Ultimately, 82 restorations were placed in permanent molars with 41 teeth in each group for an allocation ratio of 1:1(Figure 1). The primary investigator placed all the restorations after being trained by an expert to reduce technique variability (Figures 2, 3, 4, and 5). Each consecutive tooth was restored alternately using the test and control techniques. Most of the children had more than one cavity. Ten children had a single decayed tooth, five children had two decayed teeth, 11 had three decayed teeth, six had four decayed teeth and one had five decayed teeth. Appointments for children with multiple decayed teeth were scheduled in such a way that only one restoration was done on any given day to minimise the traumatic experience for the child. An assistant recorded the time taken for each procedure and the SEM criteria [12] using a stopwatch. The SEM scale is a tool used to assess pain in children. The scale measures a child's physical reactions to pain, including eyes, movements, verbal expressions and pain intensity (Table 1). This scale has been previously used and validated in India [13, 14].

In Group 1 (Carie-Care™), carious teeth were isolated using cotton rolls, and Carie-Care™ gel was applied directly to the carious lesion for 2 min. The cavity was washed, and hand instruments were used for gentle excavation. In Group 2 (ART), the mechanical method was used for caries removal, with the opening of the cavity enlarged if necessary. Following caries excavation, GIC was placed on the cavity using a cement carrier and finger pressure.

Due to school closures during the COVID-19 pandemic, follow-up assessments originally planned at 1, 6 and 12 months could not be conducted. Therefore, the earliest follow-up was performed at 18 months (Figure 1). All the restorations were evaluated by the two examiners at 18 months based on retention, marginal integrity and bulk fracture using the criteria provided by Frencken et al. in 1996 [15] (Table 2).

Data were analysed using IBM SPSS version 20. The kappa statistics for intraexaminer variability and interexaminer reliability were 0.89. The normality of the variables was assessed using the Kolmogorov–Smirnov test. Continuous variables were compared using the Mann–Whitney *U* test between the Carie-Care™ and ART groups, and a Chi-square test was conducted to compare the clinical performance of Type IX GIC restorations at 18 months between the two groups. ART Scores 0, 1 and 7 were considered successful restorations for data analysis, whilst Scores 2 and 8 were deemed failures, with no other observed scores (Table 2). A *p* value of < 0.05 was considered statistically significant.

## 3. Results

The sample consisted of 33 children (15 males and 18 females) with 82 teeth with occlusal caries. Caries was excavated using either Carie-Care™ or ART, followed by Type IX GIC restoration. Out of the 33 children, 23 had more than one restoration. The number of restorations per child ranged from 1 to 5. The mean age of the participants was 11.5 (1.5) (Table 3). The mean SEM scores and the total SEM scores were significantly higher in the conventional ART group compared to the Carie-Care™ group (*p* < 0.05). The mean time taken for caries removal in Carie-Care™ (731.15 ± 197.48 s) was significantly higher than the ART group (596.66 ± 158.96 s) (*p* < 0.001) (Table 4). At the 18-month follow-up, a total of four teeth (one in Carie-Care™ and three in the ART group) were lost to follow-up due to students leaving the school. The clinical performance evaluation of 78 teeth revealed a majority of successful restorations (80% and 76.4%) in both Carie-Care™ and ART groups, with no significant difference in the clinical performance of Type IX GIC restorations between the two groups (*p*=0.69) (Table 5).

## 4. Discussion

A randomised controlled trial was undertaken with school children to compare the effectiveness of restorations between the Carie-Care™ and ART groups after an 18-month follow-up, using ART assessment criteria. In addition, the study aimed to assess the time taken and pain experienced during caries excavation among the groups, with pain and discomfort evaluated using the SEM scale, especially beneficial for younger children facing difficulty expressing their feelings due to verbal-cognitive developmental issues [12].

In our investigation, the Carie-Care™ group required significantly more time for caries removal compared to the ART group. The mean scores for the SEM criteria were notably higher in the ART group than in the Carie-Care™ group. However, at the 18-month mark, the survival rate of Type IX GIC in the Carie-Care™ group (80.0%) was only marginally higher than in the ART group (76.4%), with no statistically significant difference. Prior studies have suggested that Carie-Care™ was less painful and more preferred by patients than ART [16–18], which aligns with our findings.

Increased time required for the Carie-Care^TM^ group as compared to the ART group reported in our study was consistent with the findings of a previous study by Rajakumar et al. [16]. The increased treatment time with Carie-Care™ was attributed to the 2-min application time in the carious cavity for softening, along with multiple applications. In addition, some children were apprehensive about the syringe used to dispense Carie-Care™, which contributed to the prolonged treatment time. Rajakumar et al. [16] also concluded in their study that pain experienced was greatest in the airotor group, followed by ART, and least in the Carie-Care^TM^ group.

To assess the effectiveness of Type IX GIC restorations at 18 months between the Carie-Care^TM^ and ART groups, we used ART assessment criteria and found no statistically significant difference between the two groups. No previous studies have directly compared the success of Type IX GIC restorations using Carie-Care^TM^ and ART techniques. Furthermore, a separate study that measured the 2-year survival rate of Class II composite resin restorations prepared by ART, with and without CMCR, also reported no difference in survival rates between the two methods [19]. A recent study that measured the bond between the resin-modified GIC (RMGIC) and residual dentin following excavation of carious dentin using Carie-Care^TM^ and conventional caries removal in primary teeth showed increased bond strength of RMGIC after Carie-Care^TM^ was used as compared to the conventional caries removal method. However, the same study also showed greater microleakage for the Carie-Care^TM^ group [20].

### 4.1. Limitations

Our study was conducted on school premises with limited resources, such as no dental chair, rubber dam or local anaesthesia. Treatment procedures were carried out using only natural light. In addition, the original follow-up timeline had to be altered due to the closure of schools during the COVID-19 pandemic. Since all the restorations were done by a single person, blinding was not possible. Blinding of the assessors was achieved due to the 18-month long follow-up time during which any recollection of patients/teeth restored would have disappeared essentially making this study a double-blind study.

In conclusion, despite similar success rates, the added advantage of reduced trauma makes using a chemomechanical agent such as Carie-Care^TM^ an attractive option for community health and school dental programs. Further studies are necessary to compare the incidence of secondary caries between the CMCR and ART groups.

## Figures and Tables

**Table 1:** Sound eye motor (SEM) scale.

Parameter	Comfortable	Mild discomfort	Moderate discomfort	Severe discomfort
Grade	1	2	3	4
Sound	No sound	Nonspecific sound	Verbal compliant and louder sound	Verbal compliant shouting and crying
Eye	No sign	Dilated eye without tears	Tears and sudden eye movement	Crying and tears all over face
Motor	Relaxed body and hand	Muscular contraction of hands	Sudden body and hand movements	Hand movement for defence and turning the head to the opposite side

**Table 2:** ART assessment criteria.

Score	Criteria
0	Present, good
1	Present, slight marginal defect for whatever reason, at any one place which is less than 0.5 mm in depth; no repair is needed.
2	Present, marginal defect for whatever reason, at any one place which is deeper than 0.5 mm but less than 1.0 mm; repair is needed.
3	Present, gross defect of more than 1.0 mm in depth; repair is needed
4	Not present, restoration has (almost) completely disappeared; treatment is needed.
5	Not present, other restorative treatment has been performed
6	Not present, tooth has been extracted
7	Present, wear and tear gradually over larger parts of the restoration but is less than 0.5 mm at the deepest point; no repair is needed
8	Present, wear and tear gradually over larger parts of the restoration which is deeper than 0.5 mm; repair is needed
9	Unable to diagnose

**Table 3:** Demographic characteristics of participants.

	Variables	*N* (%)
Sex	Male	15 (43)
	Female	18 (57)

Arch	Upper	31 (37.8)
	Lower	51 (62.2)

Age	Min-Max	9–14
	Mean (SD)	11.57 (1.5)

Tooth	15	1 (1.2)
	16	9 (11.0)
	17	4 (4.9)
	26	14 (17.1)
	27	3 (3.7)
	36	16 (19.5)
	37	12 (14.6)
	46	10 (12.2)
	47	13 (15.9)

**Table 4:** Comparison of sound and eye and motor scores between Carie-Care™ and ART groups.

	Group	Mean	Std. deviation	Significant *p* value
Sound	Carie-Care^TM^ group	1.29	0.46	0.011
	ART group	1.59	0.54	

Eye	Carie-Care^TM^ group	1.32	0.61	0.036
	ART group	1.66	0.82	

Motor	Carie-Care^TM^ group	1.41	0.59	0.030
	ART group	1.78	0.88	

SEM score	Carie-Care^TM^ group	4.02	1.38	0.011
	ART group	5.02	2.00	

Time taken for procedure (seconds)	Carie-Care^TM^ group	731.15	197.48	< 0.001
	ART group	596.66	158.96	

*Note:* Mann–Whitney *U* test.

**Table 5:** Comparison of the clinical performance of Type IX GIC restorations at 18 months between Carie-Care™ and ART groups.

Follow-up (18 months)	Carie-Care^TM^*N* (%)	ART *N* (%)	*p* value
Success (no repair required)	32 (80)	29 (76.4)	0.69
Failure (repair required)	8 (20)	9 (23.6)	

*Note:* Chi-square test.

## Data Availability

Data will be made available on request.
